# Comparative Assessment of Heart Rate Variability Obtained via Ambulatory ECG and Polar Heart Rate Monitors in Healthy Cats: A Pilot Study

**DOI:** 10.3389/fvets.2021.741583

**Published:** 2021-11-08

**Authors:** Emma K. Grigg, Yu Ueda, Ashley L. Walker, Lynette A. Hart, Samany Simas, Joshua A. Stern

**Affiliations:** ^1^Department of Population Health and Reproduction, School of Veterinary Medicine, University of California, Davis, Davis, CA, United States; ^2^Department of Clinical Sciences, College of Veterinary Medicine, North Carolina State University, Raleigh, NC, United States; ^3^Department of Medicine and Epidemiology, School of Veterinary Medicine, University of California, Davis, Davis, CA, United States; ^4^Department of Animal Science, University of California, Davis, Davis, CA, United States

**Keywords:** cat, physiological stress measures, heart rate, heart rate variability, Holter monitor, Polar H10

## Abstract

Chronic exposure to stressful environments can negatively impact cats' health and welfare, affecting behavioral, autonomic, endocrine, and immune function, as with cats in shelters. Low-stress handling practices likely improve shelter cat welfare, but data supporting improved outcomes remain limited. Cardiac activity, particularly heart rate variability (HRV), is an indicator of stress and emotional state in humans and non-human animals, tracking important body functions associated with stress responsiveness, environmental adaptability, mental, and physical health. HRV studies in cats are limited, involving mainly anesthetized or restrained cats. This pilot study tested the feasibility of obtaining HRV data from unrestrained cats, using a commercially available cardiac monitoring system (Polar H10 with chest strap), compared with data from a traditional ambulatory electrocardiogram. Simultaneous data for the two systems were obtained for five adult cats. Overall, the Polar H10 monitor assessments of HRV were lower than the true HRV assessment by ambulatory ECG, except for SDNN. Correlation between the two systems was weak. Possible reasons for the lack of agreement between the two methods are discussed. At this time, our results do not support the use of Polar H10 heart rate monitors for studies of HRV in cats.

## Introduction

Chronic exposure to stressful environments can negatively impact cats' health and welfare (1, 2). Threatening environments can be a significant factor contributing to disease, and environmental enrichment likely plays an important role in recovery of health and well-being (1, 3). Cats in shelters can be subject to high levels of environmental threat (4–6), and require medical evaluation and routine handling and care by staff. Use of low-stress handling practices has been recommended to improve the welfare of sheltered cats (7), but data supporting improved outcomes (reduced stress, leading to safer handling, better welfare, more accurate exam data) from using low-stress handling remain limited.

Heart rate variability (HRV) is an indicator of stress and emotional state in humans and non-human animals (8–13). HRV describes variation between successive heart beat intervals (R-R intervals), which reflects changes in activity of the autonomic nervous system (ANS). The ANS, composed of sympathetic and parasympathetic (vagal) branches, participates in the control of heart rate and rhythm (14). Recent research demonstrates the importance of HRV as an indicator of important body functions associated with stress responsiveness, environmental adaptability, and mental and physical health (15). In humans, decreased measures of HRV have been associated with adverse outcomes in cases of cardiovascular disease and lupus disease activity (16). Elevated stress responses, such as those caused by chronic environmental stressors (in humans, stressors present in a poor work environment), enhance the risk of cardiovascular disease (17). Assessment of HRV is common in animal research (8), and has been performed in cats on a limited basis [e.g., (18, 19)].

Psychological states such as anxiety and fear can impact the sympathovagal balance of the ANS in the absence of any detectable changes in heart or respiration rates, and changes in HRV can occur in the absence of detectable changes in heart rate (8). For example, in a study of lamb responses to aversive events, learning to prevent an aversive event was associated with elevated HRV, but was not reflected in changes in heart rate; conversely, lack of control over the aversive event was associated with decreased HRV, suggesting greater sympathetic control over cardiac activity (10, 11). Thus, measures of HRV appear to indicate ANS function more reliably than heart rate alone. Unfortunately, studies of HRV in domestic cats are limited, and often involve anesthetized or restrained cats; for example, reports used restrained cats at home vs. in the clinic (20), cats restrained in the hospital vs. freely moving at home (18), and anesthetized cats during pharmacological manipulations (19). In addition, the R-R interval used for HRV calculations is usually determined from an electrocardiogram (ECG). Use of ECGs obtained from ambulatory cats using wireless, non-invasive Holter recorders has been validated using direct recordings in cats, but analysis of the data obtained with the commercially available software is expensive and requires specific training for interpretation (18, 19, 21, 22). Preliminary studies suggest that wireless, smartphone-based heart rate sensors can generate similar data (23–25), while potentially allowing less discomfort and improved mobility for the cat.

We conducted a pilot study to test feasibility of obtaining ECG data for assessment of HRV from time domain variables, from conscious, caged cats using modifications of an inexpensive, readily available, cardiac activity monitoring system, the Polar H10 (Polar Electro Inc., Bethpage, NY). This study compared cardiac activity data collected simultaneously using two methods: Polar H10 Heart Rate Sensor units with Polar Pro soft straps to secure the electrodes around the chest of the cat, and standard ambulatory ECG recorders (Burdick H3^+^ Holter Recorder, Mortara Instrument Inc., Milwaukee, WI). Our hypothesis was that the alternate method of obtaining HRV data would provide data comparable to the Gold Standard ambulatory ECG.

## Materials and Methods

### Animals and Housing

Eight adult intact domestic cats (4 males, 4 females) between the ages of 2 and 7 years, with weights of ≥4.5 kg, and with body condition scores between 4 and 5 (on a 9-point scale) were enrolled in this pilot study. The health status of cats was assessed based on a complete veterinary exam within 6 months of enrollment, and only clinically healthy cats, with no heart disease and normal blood pressure, participated in the study. Cats used for this study are part of a university-owned campus research cat colony; cats in this colony normally live indoors in large, shared enclosures. During data collection, cats were contained in individual cages (~3 ×3 ×3 ft, containing a litter box, water, and elevated resting perch) but allowed to move freely within their cages.

### Data Collection

**Figure 1:**
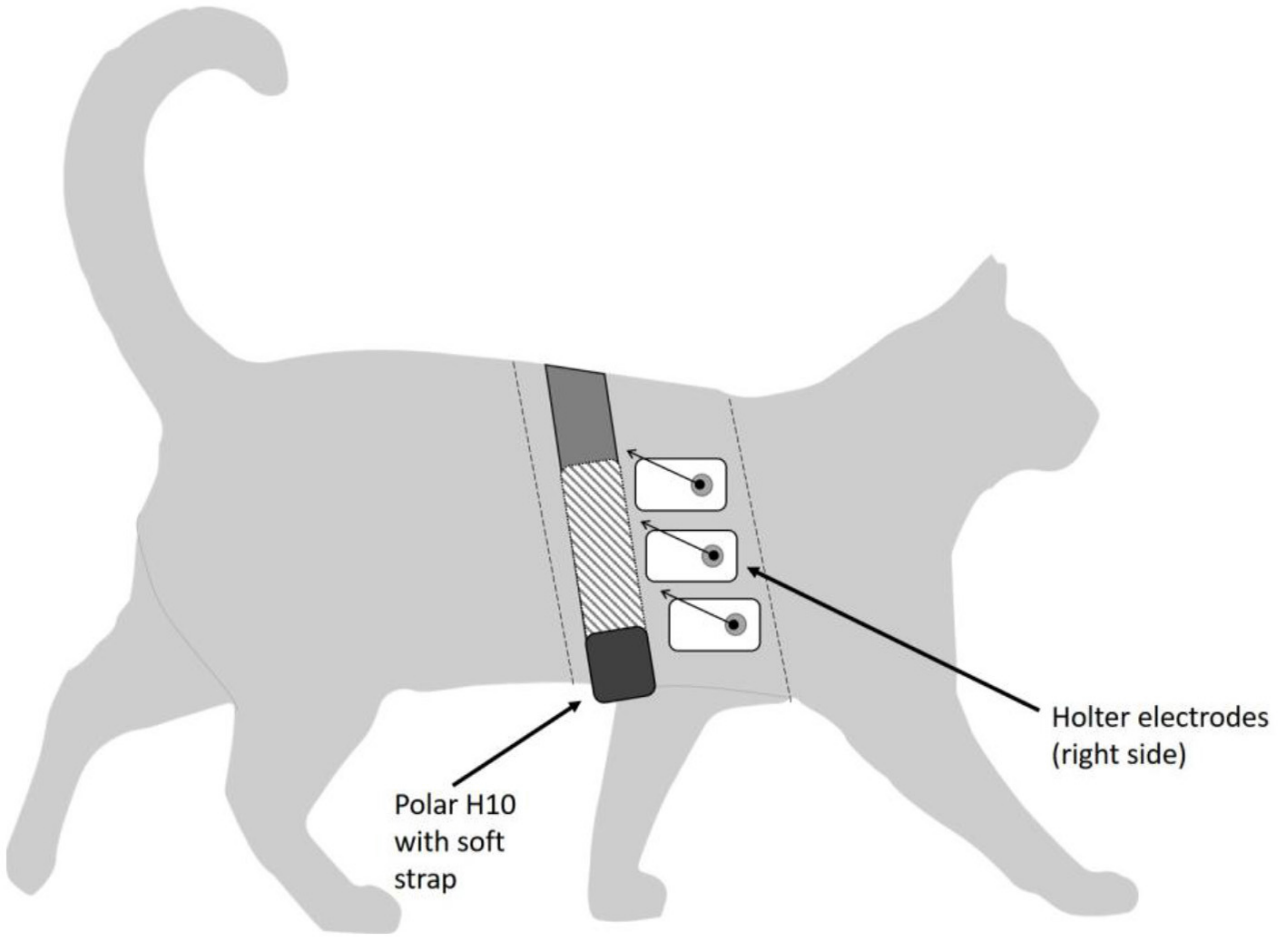
Diagram of study cat, showing placement of Holter electrodes and Polar H10 sensor with elastic strap. Dashed lines represent approximate cranial and caudal edges of the shaved area. Polar electrodes are located within the soft strap (shown as hatched area of strap). Leads on the Holter electrodes are shown as abbreviated arrows for image clarity.

Heart rate interval data were collected simultaneously from the subjects using the two different systems, the ambulatory Holter ECG monitor and the Polar H10 heart rate monitor with a Polar Pro soft strap (with two electrodes). The Polar system collected heart rate data wirelessly and transmitted data via Bluetooth® to an iPhone application (Heart Rate Variability Logger, Marco Altini: A.S.M.A.B.V.). Prior to the start of the data collection, the non-invasive cardiac sensors were fitted to the cats using low-stress handling techniques; cats were sedated for monitor application to minimize stress using sedatives butorphanol (0.2–0.3 mg/kg IM) and alfaxalone (1–3 mg/kg IM). To ensure complete contact between monitor electrodes and the subject's skin and to guarantee continuous readings from both HR monitors, a 4-inch-wide strip of fur was shaved from the cats' left and right hemithorax, and electrically conductive gel (Spectra® 360, Parker Laboratories, Fairfield, NJ) was applied between sensors and the cats' skin to optimize electrode-skin contact. First, the 5-electrode Burdick ambulatory ECG monitor with three-channel recording was attached to areas of the shaved chest that optimized the electrocardiographic signal, in a standard precordial configuration (Figure 1). Next, the Polar H10 heart rate monitor with a Polar Pro soft strap with two electrodes was attached around the cats' chest, just caudal to the ECG device (Figure 1); the sensor unit was centered ventrally, positioning an electrode on each side of the cats' thorax. Elastic bandage material (VetWrap; 3M, Maplewood, MN) was then wrapped around the cat to cover both devices and minimize slipping. Cats were allowed to recover from sedation and acclimate to the HR monitors for ~30 min, after which time cats were moved to individual cages. Simultaneous data was recorded for 6–7 h. Smartphones used to record data from the Polar monitors were located just outside the cats' cages, but as close to each cat's kennel as possible, to improve the reliability of the Bluetooth® connection. Following data collection, both monitors were removed from the cats using low-stress handling approaches; additional sedation was not required for any of the cats for monitor removal. Cats were monitored by researchers, colony facility, and veterinary staff for any adverse effects (e.g., excessive drop in HR or lack of consciousness) during sedation, and post-sedation monitoring was done on all cats on intervals of at least every 15 min. All cats were fully alert and responsive by the end of the monitoring sessions and were directly returned to the main colony. All procedures involving the cats were done under approval from the UC Davis IACUC, protocol # 21188, and in accordance with the Winn Feline Foundation's Humane Use of Animals Guidelines (March 2015).

### Heart Rate Variability Analysis

All data from the ambulatory ECG monitors was uploaded to the software analysis system, Burdick Vision 5 software (Mortara Instrument, Inc., Milwaukee, WI) following device removal from each subject. Data processing and reporting was performed as previously described (26). Briefly, analysis and interpretation of the uploaded ECG recording was made prospectively. The software analysis system automatically annotates normal and abnormal complexes, however incorrect labeling of beat type and QRS timing occurs frequently in feline recordings. Therefore, all recordings were visually inspected on a beat-by-beat basis in their entirety and all mis-labeled beats were corrected in order to accurately determine the frequency and complexity of any ectopy. QRS complexes labeled as normal but with incorrect timing, for example over the T or P wave, were manually corrected to allow for accurate and precise HRV analysis. Portions of the recordings with motion-related artifact that was significant enough to preclude accurate labeling and interpretation was labeled as artifact, discarded and not quantified for analysis. HRV was analyzed using standard time-domain techniques in accordance with published recommendations (27). All of the time-domain measures of HRV were calculated for each 1-h period using the Burdick Vision 5 ECG analysis software. The normal-to-normal (NN) intervals were calculated from one R wave to the next R wave for all sinus beats by the software. The mean, minimal, and maximal HR were recorded, based on the NN intervals. For each disclosure, the square root of the mean squared differences of successive NN interval (RMSSD), the standard deviation of all NN intervals (SDNN), the standard deviation of the average NN intervals over 5 min (SDANN), and the number of interval differences of successive NN intervals >50 ms divided by the total number of NN intervals (pNN50) were obtained. The NN intervals were also converted to the triangular index value, defined as the integral of the density distribution of the NN intervals divided by the maximal density distribution.

Polar H10 data was downloaded from the HRV Logger application into Microsoft Excel (Microsoft Corp., Redmond, WA) spreadsheets for processing and analysis. The Polar H10 data was pre-processed for analysis using the following steps: first, data was plotted (sensor readings vs. time) and visually inspected for gaps (for example, due to temporary loss of Bluetooth® connection with the smartphones); data segments with gaps were removed from the analysis, in order to ensure that comparable time periods for both data sources were being compared. As Polar H10 monitors record IBI (vs. ECG), automatic artifact correction must be used for these types of data (27, 28). As one goal of the present study was to assess a simple, user-friendly approach to use of Polar monitors for assessing heart rate and HRV in cats, and as artifacts generally present as outliers (vertical spikes) in the heart rate data, we used a straightforward outlier identification formula (29), based on removal of sensor readings >1.5 times the Interquartile Range (IQR) below 1st quartile and above 3rd quartile. This automatic outlier correction process was conducted for each cat individually. The artifact removal threshold (25%) within the HRV Logger app was not used during data collection. Cardiac data from the two data collection methods (recording start and stop times) were examined to ensure temporal synchrony of measurements during collection and prior to analysis.

From the cardiac activity data recorded during all sessions, the following time-domain cardiac parameters (27) were calculated for each cat, and for each hour of simultaneous data collection: Heart rate (HR), in beats/min (max, min, mean); root mean square of successive interbeat interval differences, RMSSD (ms); standard deviation of all interbeat intervals, SDNN (ms); and RMSSD/SDNN ratio, an index of vagosympathetic balance (12, 14, 30, 31).

### Statistical Analysis

Initial estimates of sample size for this pilot study were made according to recommendations of Ruxton and Colegrave (32), to meet or exceed sample sizes in published studies successfully able to answer similar research questions, in this case pilot studies involving HRV analyses in mammals: *n* = 2 (23), *n* = 7 (19); *n* = 8 (17, 25); *n* = 9 (33). For correlation analyses, we determined that a sample size of 8 would be necessary to achieve 80% power to detect a correlation of 0.80 between the two measures at α = 0.05 (G^*^Power 3.1.9.2). In order to compare data collected concurrently using the two alternate cardiac data collection methods, Bland Altman plots (34, 35) were constructed for all the cardiac activity parameters. Data were assessed for normality using Shapiro-Wilk tests. In addition, Pearson's correlation coefficients were calculated for each of the cardiac parameters between data collected with the Polar vs. ambulatory Holter ECG, using the hourly values of each of the cardiac parameters and including all usable hours of concurrent data collection. All statistical analyses for this study, including creation of Bland Altman plots, were done using XLSTAT (Addinsoft; NY, NY) in Microsoft Excel.

## Results

Eight cats were fitted with dual cardiac monitoring systems for this study. On two of the cats, the ambulatory Holter monitors malfunctioned, causing loss of those data. The Polar H10 sensor on a third cat suffered significant connectivity lapses on the Bluetooth® connection with the smartphone, resulting in the unreliability of those data. Loss of sensor data for these three cats meant we were not able to obtain our desired sample size. Filtering of outliers and gaps from the Polar data resulted in removal of some R-R interval data for each cat (mean loss per cat 24.5%; range 13.2–50.9%), and 6 h segments (19% of all hourly segments) were removed from the comparisons between Holter and Polar data due to unacceptably high levels of error. Usable simultaneous data for the two cardiac activity monitoring systems were available for five cats (Table 1). Once recovered from sedation and acclimated to the monitors, the cats appeared to tolerate the presence of the monitors well, throughout data collection. No adverse events occurred with any of the cats.

**Table 1:** Cats used for Polar-Holter cardiac data comparisons for this study, showing mean, min, and max HR recorded for the duration of data collection for both methods (Polar H10 filtered data, and the Holter data).

**Cat ID**	**Age (years)**	**Kg**	**Sex**	**Polar H10**		**Ambulatory ECG (Holter)**		***n* (hours)**
				**HR Mean ± SD**	**HR Min/Max**	**HR Mean ± SD**	**HR Min/Max**	
13099	7	5.3	F	202.3, 9.6	188/217	205.3, 15.1	188/235	6
14011	6	5.4	F	160.3, 24.3	127/184	234.3, 4.8	230/241	3
14077	6	5.3	F	203, 6.5	193/213	212.8, 20.5	193/252	5
16010	4	5.2	M	188.2, 9.3	175/204	190.2, 9.9	179/204	6
18013	2	5.2	M	183.4, 15.0	155/195	223.4, 10.0	210/241	5

*Note that detailed data for all HRV variables by hour of data collection is available as Supplementary Materials*.

Summary data, by hour of simultaneous data collection, for all cats are available in Supplementary Materials. Mean HRs recorded for the duration of data collection for both methods—Polar H10 filtered data, and the Holter data—are shown in Table 1. Normality of the data for all cats was confirmed (*p* > 0.64 for all cats).

**Figure 2:**
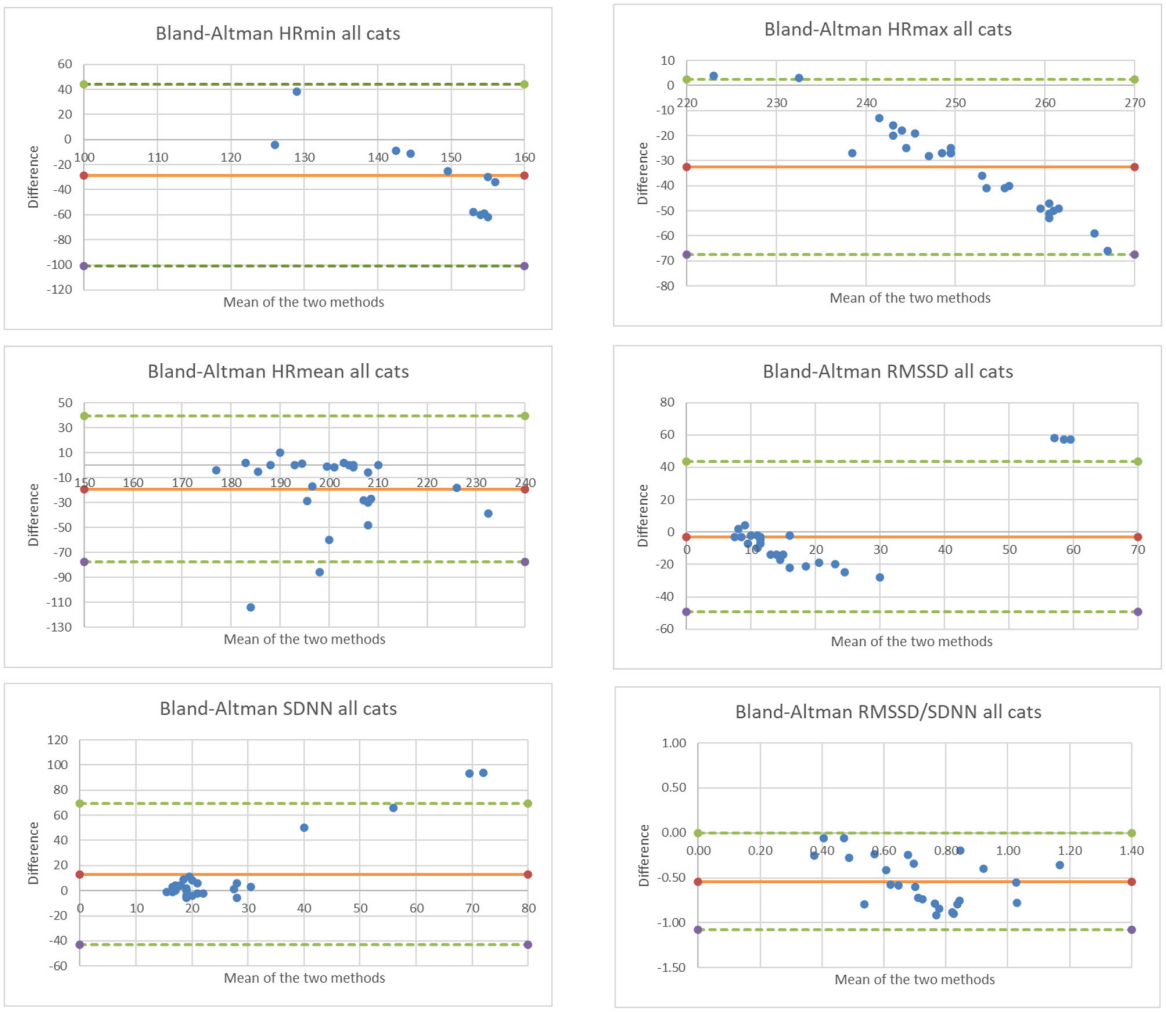
Bland Altman plots comparing data collected for 5 cats and for all cardiac parameters using the two methods. Note that the x-axis represents the mean measurement (Holter and Polar H10), and the y-axis represents the difference between measurements (Polar H10 minus Holter).

The limits of agreement between the two cardiac data collection methods are shown in the Bland Altman plots for all cardiac parameters (Figure 2). Mean difference between the two methods is shown as a solid orange horizontal line on all plots; a mean difference = 0 (i.e., *y* = 0) would indicate strong agreement between the two methods. Upper and lower limits of agreement are shown as dashed green horizontal lines on the Bland Altman plots (Figure 2). Overall, the Polar monitor readings (filtered) were lower than the Holter monitor readings, except for SDNN, as follows:

- HR_minimum_ average difference −28.5 bpm
- HR_maximum_ average difference −32.6 bpm
- HR_mean_ average difference −19.3 bpm
- RMSSD average difference −2.9 ms
- SDNN average difference +13.0 ms
- RMSSD/SDNN average difference −0.5.

Correlations between Polar data (raw and filtered) and the Holter data for all cardiac parameters and cats are shown in Table 2. Strength of the correlations varied widely between cats, and by cardiac parameter; only some of the correlations were statistically significant, suggesting a lack of consistent agreement between the two methods.

**Table 2:** Correlations between Polar H10 data (raw data, and data filtered using outlier removal methods described in the text) and the ambulatory ECG (Holter) data for all cardiac parameters and all cats used for data analysis.

**Cat ID**		**HR (min)**		**HR (max)**		**HR (mean)**		**RMSSD**		**SDNN**		**RMSSD/SDNN**	
		**(Raw)**	**(Filtered)**	**(Raw)**	**(Filtered)**	**(Raw)**	**(Filtered)**	**(Raw)**	**(Filtered)**	**(Raw)**	**(Filtered)**	**(Raw)**	**(Filtered)**
13099	*r*	0.203	0.619	**0.858**	**0.858**	−0.509	**0.946**	0.662	0.326	0.366	0.162	−0.302	0.290
	*p*	0.70	0.19	0.029	0.029	0.30	0.004	0.15	0.53	0.48	0.76	0.56	0.58
14011	*r*	**0.999**	**0.999**	−0.500	−0.500	−0.917	−0.917	0.681	0.982	−0.160	0.577	0.922	0.716
	*p*	0.005	0.005	0.67	0.67	0.26	0.26	0.52	0.12	0.90	0.61	0.25	0.50
14077	*r*	−0.336	0.272	0.671	0.671	**0.908**	**0.888**	**−0.945**	−0.466	−0.635	−0.450	0.210	0.637
	*p*	0.52	0.60	0.15	0.15	0.012	0.012	0.004	0.35	0.18	0.37	0.70	0.17
16010	*r*	**−0.912**	**−0.819**	0.463	0.463	0.547	0.624	0.350	**0.883**	0.095	0.610	0.522	**0.855**
	*p*	0.011	0.046	0.36	0.36	0.26	0.19	0.50	0.020	0.86	0.20	0.29	0.030
18013	*r*	0.444	0.250	0.706	0.706	−0.687	−0.681	0.824	−0.214	0.028	−0.318	−0.722	−0.785
	*p*	0.45	0.69	0.18	0.18	0.20	0.21	0.09	0.73	0.96	0.60	0.17	0.12

*For each correlation, the correlation coefficient (r) and p-values are shown. Significant correlations are shown in bold font*.

## Discussion

Although Polar HR monitoring systems are well-regarded for data accuracy and reliability, and have been used in a number of published research studies on dogs (12, 30, 36, 37), there was not strong and consistent agreement between data from the Polar H10 chest-strap sensors and the Holter ECG recorders for the cats in our study. Possible reasons for this lack of agreement are listed below. At this time, these results would not support the use of Polar H10 HR monitors with chest strap attachment for studies of HRV in free-ranging cats.

Mean HR during data collection for these cats ranged from 160.3 to 234.3 bpm, with most cats averaging over 200 bpm (Table 1). For comparison, resting HRs in non-stressed cats range from 120 to 140 bpm (38), with stressed cats' HR reaching up to 220 bpm. Abbott (18) reported mean HRs for 16 healthy young cats, noting that HR varied with location and stress level of the animals: mean HR when restrained (measured via electrocardiogram) was 187 ± 25 bpm; mean HR while resting in the veterinary clinic (measured via radiotelemetry device) was 150 ± 23 bpm; and mean HR at home (radiotelemetry device) was 132 ± 19 bpm. Regardless of method of cardiac data collection, mean HRs of cats in this study were high, suggesting that these cats were stressed by dual monitor presence and/or their containment in the holding cages (separate from their normal living quarters), despite lack of obvious behavioral signs of stress. This should not have impacted comparisons, however, as presumably readings from both monitoring systems on the same cat would have been similarly impacted.

The small size of the study animals made concurrent fitting with both ambulatory Holter monitors and Polar H10 monitor chest straps challenging; the influence of the close proximity (Figure 1) on data quality is unknown. In studies of other smaller species such as rodents (39, 40), more invasive methods (i.e., implanted transmitters) are sometimes used to obtain good quality HRV data. The Polar H10 with chest strap was designed for use on a human chest; distance between the electrodes located within the chest strap may have resulted in suboptimal placement of the electrodes on the cat's thorax. However, as artifacts were discarded from the data as described above, slipping or shifting of the electrodes, while potentially impacting ECG configuration, should not impact the R-R interval data used in these analyses. Although ambulatory Holter monitors alone have been used successfully on cats (39), Von Borell et al. (8) cautioned in their review that long-term HRV monitoring should only be measured while animals are stationary, with at most minimal (or unvarying) activity. Within the confines of their individual cages, the cats in this study were free to move at will during data collection.

Bluetooth® connectivity issues between the Polar H10 sensors and the smartphones were common; for some cats, the connection between sensor and tag was lost seemingly at random intervals, and had to be manually reconnected by the researcher using the HRV logger app on the phones. As a result, despite periodic checks during the 7-h data collection sessions, data was lost due to dropped connections, which reduced the sample size (amount of continuous, synchronous Polar data) available for analysis. The Polar H10 tag on one cat suffered sufficient connectivity lapses that data from that cat was considered unreliable and was removed from the analysis. Anecdotally and based on recent experience using Polar H10 monitors on dogs (Grigg and Hart, unpublished data), this issue may be exacerbated by the presence of multiple Polar monitors and smartphones within a relatively small area. The colony housing space utilized in this study measured ~15′ × 25′, and housed 4 monitored cats at a time.

It is important to note that, if either of the two problems above were causing the lack of agreement between the two cardiac monitoring systems, it may be that—if Polar monitors were used alone (w/o concurrent Holter monitoring) and/or without other Polar monitors nearby—they may function reliably. Our study design did not allow us to investigate that possibility. In addition, two of the Holter monitors suffered a malfunction prior to data download, resulting in loss of all Holter data for two study cats.

Finally, although guidelines for prospective analysis of the Holter data were followed carefully and completely (27), and every effort made to remove anomalous readings from the Polar data, it may be that user error in pre-processing or analyses of the Holter and/or Polar cardiac data impacted our results. No trend removal was done for the Polar data. And, although our artifact removal method did, overall, appear to improve the accuracy of the Polar sensor data (see Supplementary Materials), the artifact removal was automatic and therefore may have removed valid IBI data.

In conclusion, this study did not find sufficient agreement between standard ambulatory ECG and Polar monitor HRV data to suggest the utility of Polar H10 monitors in future feline HRV studies. HRV analyses have been used to assess emotional state (reflecting stress levels and welfare) of a number of mammalian species (e.g., horses, cows, dogs, and rats) under different handling, housing, training, and management protocols and routines (30, 38, 41–45). Physical restraint and manipulation during physical examination alter physiological parameters such as HR and HRV (20). Identifying a method of accurately and reliably determining HR and HRV in unrestrained cats during handling, daily husbandry procedures, among others, at a reasonable cost, would provide researchers and veterinarians with a valuable tool to assess stress levels associated with these procedures. Additionally, this method could be used to measure efficacy of interventions designed to reduce stress and increase welfare (such as low-stress handling procedures) of cats housed in shelters, clinics, and homes. Further evaluation of alternate methodologies for processing Polar monitor HRV data is warranted, as it remains our goal to identify an accessible, user-friendly, but reliable method to obtain HRV data on freely-moving domestic cats.

## Data Availability Statement

The datasets presented in this study can be found in online repositories. The names of the repository/repositories and accession number(s) can be found at: https://figshare.com/s/eda5ed5d9249e57f7d79.

## Ethics Statement

The animal study was reviewed and approved by UC Davis Institutional Animal Care and Use Committee (IACUC).

## Author Contributions

EG conceived of the study, obtained funding and ethics approval, participated in data collection, performed data analysis, and drafted the manuscript. YU, JS, and AW assisted in study design, ethics approval, animal procedures, data collection, data analysis, and manuscript review and editing. SS generated the Holter data. LH reviewed and edited the funding proposal and manuscript. All authors contributed to the article and approved the submitted version.

## Funding

This work was funded by the Winn Feline Foundation, Grant Number W19-012, and the UC Davis Center for Companion Animal Health, Grant Number V451WF4. The UC Davis Library provides partial funds to support open access publication fees.

## Conflict of Interest

The authors declare that the research was conducted in the absence of any commercial or financial relationships that could be construed as a potential conflict of interest.

## Publisher's Note

All claims expressed in this article are solely those of the authors and do not necessarily represent those of their affiliated organizations, or those of the publisher, the editors and the reviewers. Any product that may be evaluated in this article, or claim that may be made by its manufacturer, is not guaranteed or endorsed by the publisher.
