# Supplementary material for: Comparative Assessment of Heart Rate Variability Obtained via Ambulatory ECG and Polar Heart Rate Monitors in Healthy Cats: A Pilot Study
Source: Front Vet Sci. 2021 Nov 8;8:741583. doi: 10.3389/fvets.2021.741583 (PMC8606523; doi:10.3389/fvets.2021.741583)
Supplement: Supplementary file 1 [file Data_Sheet_1.DOCX]

**Supplementary Material:** **Hourly summaries of cardiac activity data for all cats used in the comparison of Holter vs. Polar H10 data;** note that for the Polar monitors, both raw and filtered (see text for methods) data are shown.

| **cat colony ID** | **Hour** | **HR min polar (raw)** | **HR min polar (filtered)** | **HR min Holter** | **HR max polar (raw)** | **HR max polar (filtered)** | **HR max Holter** | **HR mean polar (raw)** | **HR mean polar (filtered)** | **HR mean Holter** | **RMSSD polar (raw)** | **RMSSD polar (filtered)** | **RMSSD Holter** | **SDNN polar (raw)** | **SDNN polar (filtered)** | **SDNN Holter** | **RMSSD/ SDNN polar (raw)** | **RMSSD/ SDNN polar (filtered)** | **RMSSD/ SDNN Holter** |
| --- | --- | --- | --- | --- | --- | --- | --- | --- | --- | --- | --- | --- | --- | --- | --- | --- | --- | --- | --- |
| 16010 | 1 | 119 | 140 | 170 | 236 | 236 | 276 | 204 | 204 | 202 | 28 | 5 | 27 | 60 | 16 | 22 | 0.47 | 0.31 | 1.23 |
|  | 2 | 121 | 139 | 173 | 235 | 235 | 262 | 188 | 188 | 205 | 14 | 8 | 29 | 36 | 21 | 23 | 0.39 | 0.38 | 1.26 |
|  | 3 | 121 | 137 | 162 | 236 | 236 | 263 | 188 | 183 | 188 | 23 | 11 | 30 | 44 | 31 | 25 | 0.52 | 0.35 | 1.20 |
|  | 4 | 122 | 139 | 150 | 235 | 235 | 253 | 188 | 184 | 182 | 32 | 12 | 37 | 44 | 32 | 29 | 0.73 | 0.38 | 1.28 |
|  | 5 | 148 | 148 | 110 | 225 | 225 | 252 | 178 | 175 | 179 | 59 | 16 | 44 | 51 | 25 | 31 | 1.16 | 0.64 | 1.42 |
|  | 6 | 121 | 138 | 147 | 235 | 235 | 251 | 196 | 195 | 185 | 111 | 13 | 33 | 95 | 28 | 27 | 1.17 | 0.46 | 1.22 |
| 18013 | 2 | 121 | 124 | 128 | 235 | 235 | 286 | 154 | 155 | 241 | 26 | 9 | 14 | 74 | 65 | 15 | 0.35 | 0.14 | 0.93 |
|  | 3 | 121 | 124 | 182 | 234 | 234 | 287 | 193 | 193 | 223 | 17 | 6 | 9 | 44 | 24 | 18 | 0.39 | 0.25 | 0.50 |
|  | 4 | 121 | 124 | 184 | 235 | 235 | 276 | 195 | 195 | 222 | 25 | 7 | 10 | 54 | 20 | 16 | 0.46 | 0.35 | 0.63 |
|  | 5 | 123 | 125 | 184 | 235 | 235 | 284 | 193 | 193 | 221 | 19 | 9 | 7 | 34 | 24 | 16 | 0.56 | 0.38 | 0.44 |
|  | 6 | 123 | 124 | 186 | 233 | 233 | 261 | 181 | 181 | 210 | 16 | 11 | 7 | 29 | 25 | 14 | 0.55 | 0.44 | 0.50 |
| 13099 | 1 | 119 | 153 | 116 | 237 | 237 | 262 | 170 | 217 | 235 | 84 | 6 | 16 | 112 | 17 | 17 | 0.75 | 0.35 | 0.94 |
|  | 2 | 119 | 165 | 182 | 236 | 236 | 255 | 209 | 210 | 210 | 51 | 6 | 13 | 43 | 15 | 16 | 1.19 | 0.40 | 0.81 |
|  | 3 | 120 | 166 | 172 | 235 | 235 | 248 | 198 | 199 | 200 | 48 | 9 | 11 | 44 | 20 | 16 | 1.09 | 0.45 | 0.69 |
|  | 4 | 159 | 159 | 169 | 225 | 225 | 221 | 188 | 188 | 188 | 56 | 15 | 17 | 49 | 20 | 18 | 1.14 | 0.75 | 0.94 |
|  | 5 | 122 | 156 | 176 | 236 | 236 | 263 | 203 | 205 | 205 | 48 | 10 | 13 | 44 | 19 | 15 | 1.09 | 0.53 | 0.87 |
|  | 6 | 170 | 170 | 171 | 234 | 234 | 231 | 195 | 195 | 194 | 47 | 10 | 12 | 51 | 18 | 15 | 0.92 | 0.56 | 0.80 |
| 14011 | 2 | 66 | 66 | 170 | 236 | 236 | 286 | 127 | 127 | 241 | 344 | 88 | 31 | 314 | 89 | 23 | 1.10 | 0.99 | 1.35 |
|  | 3 | 72 | 72 | 176 | 237 | 237 | 286 | 170 | 170 | 230 | 314 | 86 | 28 | 298 | 119 | 25 | 1.05 | 0.72 | 1.12 |
|  | 5 | 94 | 94 | 199 | 237 | 237 | 284 | 184 | 184 | 232 | 310 | 87 | 30 | 289 | 116 | 23 | 1.07 | 0.75 | 1.30 |
| 14077 | 1 | 120 | 155 | 174 | 236 | 236 | 295 | 148 | 213 | 252 | 186 | 8 | 15 | 92 | 23 | 14 | 2.02 | 0.35 | 1.07 |
|  | 2 | 122 | 159 | 181 | 235 | 235 | 271 | 202 | 204 | 206 | 118 | 6 | 23 | 48 | 17 | 21 | 2.46 | 0.35 | 1.10 |
|  | 3 | 119 | 155 | 177 | 234 | 234 | 300 | 198 | 205 | 211 | 151 | 6 | 20 | 63 | 18 | 22 | 2.40 | 0.33 | 0.91 |
|  | 4 | 126 | 156 | 162 | 233 | 233 | 253 | 192 | 193 | 193 | 109 | 8 | 22 | 37 | 20 | 22 | 2.95 | 0.40 | 1.00 |
|  | 5 | 120 | 156 | 164 | 233 | 233 | 274 | 197 | 200 | 202 | 124 | 7 | 22 | 49 | 19 | 19 | 2.53 | 0.37 | 1.16 |
